# Obstructive Cholestasis in IL12RB1 Deficiency: A Case Report of an Unusual Hepatobiliary Presentation in Mendelian Susceptibility to Mycobacterial Disease

**DOI:** 10.1155/crpe/8888681

**Published:** 2026-08-02

**Authors:** Nabeel Ahmad, Hooria Rehman, Muhammad Huzaifa Ameer, Syed Junaid Haide, Umer Abdullah, Bilal Aslam, Muneeb Saifullah, Hamza Aka Khail

**Affiliations:** ^1^ Department of Pediatrics, Lahore General Hospital, Lahore, Pakistan; ^2^ Department of Medicine, King Edward Medical University, Lahore, Pakistan, kemu.edu.pk; ^3^ Department of Medicine, Kateb University, Kabul 1006, Afghanistan, duhs.edu.pk

**Keywords:** biliary stricture, cholestasis, genetic pleiotropy, granulomatous lymphadenitis, IL12RB1 mutation, immunodeficiency, Mendelian susceptibility to mycobacterial disease, recurrent infections

## Abstract

While *IL12RB1* deficiency is classically associated with susceptibility to mycobacterial and *Salmonella* infections, its clinical spectrum may be broader than currently documented literature. Timely recognition of atypical pleiotropic presentations can contribute to earlier diagnosis, more tailored interventions, and eventually better patient outcome. However, current literature lacks reports of hepatic involvement such as biliary strictures or intrahepatic cholestasis in *IL12RB1*‐deficient patients. This case exemplifies the diagnostic odyssey of a child with a rare genetic anomaly manifesting as two seemingly unrelated pathophysiologies; the diagnosis was established through genetic testing after exhaustive evaluation excluded infectious, metabolic, and autoimmune etiologies. This case raises the possibility of atypical hepatobiliary manifestations associated with *IL12RB1* deficiency. Early genetic testing should be integrated into the diagnostic pathways with undiagnosed cholestasis, and clinicians should remain vigilant for systemic manifestations beyond the classical infectious profile.

## 1. Introduction

MSMD is caused by inherited defects affecting the IL‐12/IL‐23–IFN‐γ axis, and *IL12RB1* deficiency impairs IL‐12 and IL‐23 signaling, therefore resulting in defective IFN‐γ production by T cells and natural killer cells [1]. IL‐12Rβ1–deficient patients classically present in infancy or early childhood with disseminated infections after Bacillus Calmette–Guérin (BCG) vaccination, environmental mycobacteria, or *Mycobacterium tuberculosis*. Salmonellosis occurs in a minority of MSMD patients, and candidiasis happens even less frequently [2]. Burden of IL‐12Rβ1–mediated MSMD can be underscore both the global reach and the South Asian, particularly in areas where BCG is routinely given and consanguineous marriages are common by the increased reporting of this rare diagnosis [3].

The clinical spectrum of MSMD is notably heterogeneous, owing to variable genetic mutations across multiple loci and the phenomenon of incomplete penetrance rendering the presentation of this deficiency as markedly variable, ranging from localized infections to life‐threatening disseminated disease, depending on the specific molecular defect and environmental exposures [3].

Although disseminated mycobacterial disease is the hallmark of IL‐12Rβ1 deficiency, hepatic involvement has been reported [4]. However, cholestatic liver injury and biliary tract abnormalities have not been widely recognized in MSMD. Our case report discusses an 8‐year‐old IL‐12Rβ1–deficient boy from Pakistan developing marked cholestasis with extrahepatic biliary stricture. These nonclassical features may broaden the known spectrum of *IL12RB1* mutations and suggest that unexplained cholestasis or biliary obstruction in endemic regions should prompt consideration of MSMD.

## 2. Case Presentation

**FIGURE 1:**
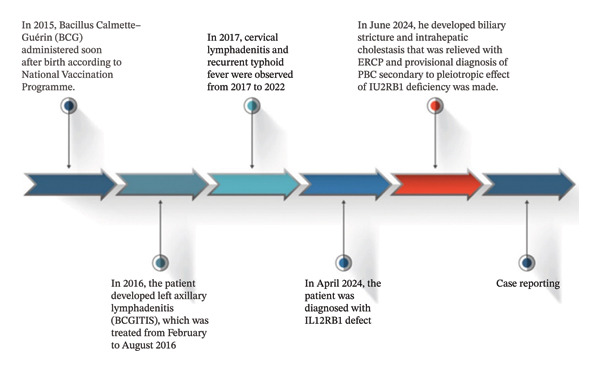
This figure delineates the patient’s clinical trajectory, highlighting key chronological milestones of medical intervention.

**FIGURE 2:**
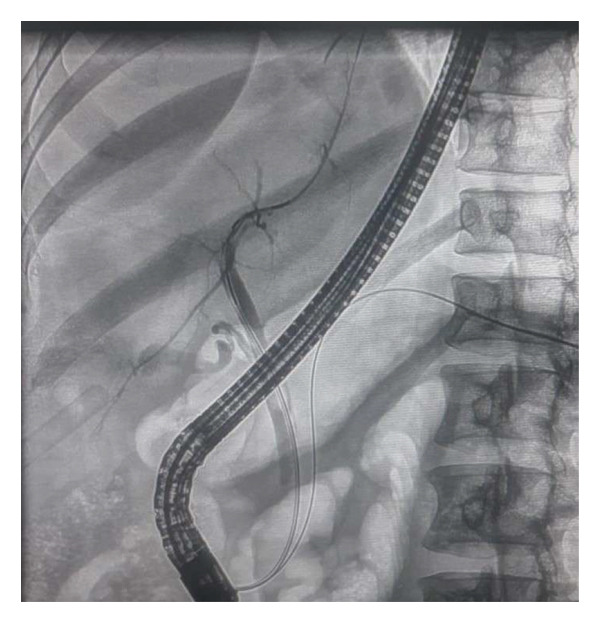
This figure shows cannulation of CBD and PD.

**FIGURE 3:**
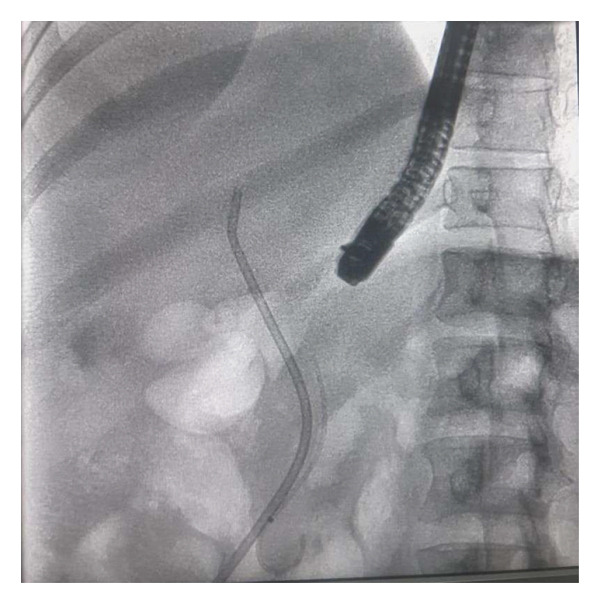
This figure shows CBD and PD stent.

An 8‐year‐old male patient, vaccinated with BCG at birth, had a history of mycobacterial lymphadenitis at the age of 1 year and recurrent febrile illnesses with documented salmonellosis and *Bacillus* species on blood cultures. The patient received multiple courses of intravenous and oral antibiotics during recurrent episodes of enteric fever and *Bacillus* bacteremia based on culture sensitivity patterns. The patient’s clinical course and major interventions are summarized in Figure 1. He was initially diagnosed with disseminated tuberculosis involving the lungs and lymph nodes based on compatible clinical findings, histopathological evidence of caseating granulomatous inflammation on excisional biopsy, and acid‐fast bacilli (AFB) isolation from gastric aspirate samples. Anti‐tuberculous therapy was subsequently initiated. Apart from tuberculosis, he also had recurrent culture‐proven enteric fever on many occasions that would always respond to oral and intravenous antibiotics. The patient had repeated febrile episodes with documented *Bacillus* sepsis, hepatic abscess, and enteric fever in the timeline. Extensive workup to rule out inborn error of immunity including immunological investigations was performed, and the results are summarized in Table 1. The results of immunological investigations are summarized in Table 1. Key findings included markedly elevated serum IgE levels and mildly reduced CD56+ natural killer cell counts, while other immunological parameters were within age‐appropriate reference ranges. Genetic testing for inborn errors of immunity performed in April 2024 identified a homozygous 4‐base pair insertion in exon 9 of the *IL12RB1* gene (chr19:18183152 T > TTCCA), resulting in a frameshift and premature protein termination 32 amino acids downstream of codon 264 (p.Gln264Leufs^∗^32; NM_005535.3). The variant was absent from population databases including gnomAD, ExAC, and 1000 Genomes, and the affected region was conserved across species. Based on its predicted frameshift effect, premature protein truncation, and consistency with the patient’s clinical phenotype, the variant was classified as likely pathogenic. The patient was a product of consanguineous marriage. There was no known family history of recurrent severe infections, mycobacterial disease, unexplained childhood deaths, or any diagnosed inborn errors of immunity. Sanger sequencing for parental carrier status analysis could not be performed. After his genetic testing, he presented again In June 2024, with acute febrile illness and significant unintentional weight loss, and the patient had also developed scleral icterus 20 days back with his laboratory evaluation demonstrating mixed hepatocellular–cholestatic liver dysfunction with direct hyperbilirubinemia and elevated liver enzymes (Table 1). Serum total protein was reduced (5.7 g/dL) with preserved serum albumin levels (3.8 g/dL). Coagulation parameters including activated partial thromboplastin time, prothrombin time, and international normalized ratio remained within normal limits. Complete blood count revealed anemia, leukocytosis with neutrophilia, eosinophilia, monocytosis, and relative lymphopenia (Table 1). Peripheral blood smear demonstrated normocytic normochromic erythrocytes with target cells and leukocytosis with left shift. Viral hepatitis PCR testing, *Cytomegalovirus* (CMV), and Epstein–Barr virus (EBV) serologies were negative. Additional evaluation for IgG4‐related disease, bile acid profiling, *Cryptosporidium*, microsporidia, and granulomatous cholangitis could not be completed due to limited diagnostic availability, which represents a limitation of this report. Targeted metabolic screening was also performed, including urine analysis for succinylacetone to evaluate for tyrosinemia type I and cystine‐related metabolites, both of which were within normal limits. An abdominal ultrasound scan showed no residual or recurrent hepatic abscesses, normal imaging of other major abdominal or pelvic viscera, mildly distended small bowel loops, and tight structural narrowing at middle and distal parts of CBD resulting in moderate extrahepatic biliary dilatation with no signs of cholangitis, choledochal cyst, or choledocholithiasis. MRCP was suggestive of biliary stricture in long segment of CBD with no fusiform dilatation having intrahepatic cholestasis (Figure 2). In this occasion, ERCP was done for biliary stenting; it revealed bulging ampulla; pancreatic and biliary cannulation was done. Cholangiogram obtained showed a stricture involving the pancreatic portion of CBD with retrograde ductal dilatation. Pancreatogram showed mildly prominent PD (Figure 3). Small‐access sphincterotomy was made followed by stricture dilatation with a 10‐Fr Soehendra dilator. A 10‐Fr x 7‐cm straight plastic stent was placed across CBD, and a 5‐Fr x 7‐cm single pigtail plastic stent was placed in PD. Free flow of bile was seen, and the patient was discharged on Day 3 post procedure with his presenting complaints settled and was placed on strict follow‐up.

**TABLE 1:** Reference ranges are representative pediatric reference intervals for children aged approximately 8 years and may vary slightly between laboratories.

Investigation	Result	Age‐specific reference range (8 years)
Hematology		
Hemoglobin (g/dL)	9.7	11.5–15.5
White blood cell count (× 10^9^/L)	35.6	4.5–13.5
Lymphocyte subsets		
CD3+ T lymphocytes (/µL)	2219	1200–2600
CD4+ T lymphocytes (/µL)	1090	650–1500
CD8+ T lymphocytes (/µL)	1089	370–1100
CD19+ B lymphocytes (/µL)	294	200–1600
CD56+ NK cells (/µL)	181	200–700
Humoral immunity		
Serum IgA (mg/dL)	90.6	34–305
Serum IgE (IU/mL)	14,014	< 90
C3 (mg/dL)	96	90–180
C4 (mg/dL)	14	10–40
Functional and microbiological studies		
Dihydrorhodamine (DHR) assay	Normal neutrophil oxidative burst	Normal oxidative burst
HIV 4th generation assay	Nonreactive	Nonreactive
Anti‐*Candida* antibodies	Positive	Negative
Anti‐*Escherichia coli* antibodies	Positive	Negative
Tissue culture	*Bacillus* species isolated	No pathogenic growth
Genetic testing		
Whole exome sequencing	Homozygous likely pathogenic IL12RB1 variant detected	No pathogenic variant detected
Hepatobiliary investigations		
Total bilirubin (mg/dL)	9.3	0.2–1.2
ALT (U/L)	97	< 40
AST (U/L)	57	< 40
GGT (U/L)	65	7–32
Total protein (g/dL)	5.7	6.0–8.0
Albumin (g/dL)	3.8	3.5–5.0

## 3. Discussion

MSMD is defined by a susceptibility to mycobacteria, including specific strains of BCG vaccine and environmental mycobacteria, leading to disseminated tuberculosis infection on BCG administration as well as recurrent infection. MSMD is also associated with high mortality rates (out of 17 cases, mortality was reported to be 32.3%) as documented [5]. Also, multiple instances of salmonellosis [6] and *Bacillus* sepsis, candidiasis, along with granulomatous lymphadenitis initially treated as tuberculosis in this case as MSMD predisposes individuals to these otherwise poorly pathogenic organisms. According to multiple reports [7, 8], children born to consanguineous parents have higher risk of manifestations of inborn error of immunity. AR forms are more frequent forms of inherited mutations. Studies reveal consanguineous marriages lead to more founder effect which ultimately results in high rate of autosomal recessive mutation inheritance patterns in South Asia including Pakistan [9]. Patients born in regions with high rates of consanguinity may carry a greater risk of autosomal recessive inborn errors of immunity. A study reveals MSMD in individuals with mutated *IL12Rb1* gene are more susceptible to disseminated BCG vaccine tuberculosis than those with intact *IL12RB1* receptor as PPD specific INF‐gamma release was compromised [9]. The IL‐12/IL‐23–IFN‐γ signaling axis plays a critical role in host defense against intracellular pathogens by promoting T‐cell and natural killer cell activation and enhancing macrophage‐mediated immunity. Although hepatobiliary involvement has been described in certain inborn errors of immunity, cholestatic disease with extrahepatic biliary stricture remains an unusual finding in *IL12RB1* deficiency [10]. While a direct causal relationship could not be established in our single case, this presentation raises the possibility of atypical hepatobiliary manifestations associated with dysregulated IL‐12/IFN‐γ signaling. Experimental studies suggest that inflammatory cytokine signaling pathways may contribute to bile duct injury and obstruction in certain cholestatic disorders, while genetic susceptibility involving IL‐12 signaling components has also been described in immune‐mediated cholestatic disorders [11]. In this context, the coexistence of genetically confirmed *IL12RB1* deficiency and unexplained biliary stricture in our patient may raise the possibility of a broader hepatobiliary phenotype associated with impaired IL‐12/IFN‐γ signaling.

## 4. Conclusion

Diagnosing *IL12RB1* mutations should be considered in patients with recurrent, severe, or persistent TB infection especially after obtaining the BCG vaccination. Taking a detailed infection, immunization, and family history together with conducting immunological and genetic tests will identify MSMD early enough to avoid complications from the disease.

To ensure timely and accurate diagnosis of MSMD and related inborn errors of immunity, it is essential that healthcare providers, at all levels, receive structured awareness campaigns and practical capacity‐building efforts enabling clinicians to recognize the early signs, patterns, and diagnostic pathways for these often‐overlooked conditions. By supporting earlier identification and referral, such initiatives can help reduce unnecessary treatments, minimizing the disease and treatment‐related complications. Most importantly, they can significantly improve the long‐term health and quality of life for affected children and their families. In resource‐limited settings, basic immunological screening and genetic testing should be incorporated into routine pediatric evaluations especially when encountered with atypical presentations or persistent infections.

## Funding

The authors have nothing to report.

## Ethics Statement

Ethical approval is taken.

## Consent

Written informed consent was obtained from the patient’s parent for publication of this case report and any accompanying images. A copy of the written consent is available for review by the Editor‐in‐Chief of this journal.

## Conflicts of Interest

The authors declare no conflicts of interest.

## Data Availability

Any data not published within the article will be made available in anonymized form on request from any qualified investigator.
